# Adjunctive middle meningeal artery embolization with low-viscosity liquid embolic agents for acute epidural hematoma: technical considerations and a case series

**DOI:** 10.3389/fneur.2026.1896356

**Published:** 2026-08-12

**Authors:** Hualong Shen, Yaohua Qian, Jinqing Hu, Tiantian Gao, Minfang Sheng, Xiaoqing Qian, Jili Chen, Yulei Ding, Xiuping Jiang, Suqin Dong, Haixia Liu, Junfeng Lu, Lei Hao, Tingting Xu, Dongmei Xia, Jinxia Luo, Yuling Cai, Jinghua Chen

**Affiliations:** 1 Department of Neurosurgery, Taicang TCM Hospital Affiliated to Nanjing University of Chinese Medicine, Taicang, Jiangsu, China; 2 Department of Neurosurgery, Ruijin Hospital, Shanghai Jiaotong University School of Medcine, Shanghai, China; 3 Department of Anesthesiology, Taicang TCM Hospital Affiliated to Nanjing University of Chinese Medicine, Taicang, Jiangsu, China; 4 Department of Operating Room, Taicang TCM Hospital Affiliated to Nanjing University of Chinese Medicine, Taicang, Jiangsu, China; 5 Department of Radiology, Taicang TCM Hospital Affiliated to Nanjing University of Chinese Medicine, Taicang, Jiangsu, China

**Keywords:** acute epidural hematoma, endovascular treatment, hematoma expansion, low-viscosity liquid embolic agents, middle meningeal artery embolization, traumatic intracranial hemorrhage

## Abstract

**Objective:**

To assess the feasibility, safety, and short-term outcomes of adjunctive middle meningeal artery (MMA) embolization using low-viscosity liquid embolic agents (1:1 diluted Onyx/DMSO and 1:9 diluted Glubran 2/Ethiodized Poppyseed Oil Injection) for acute epidural hematoma (AEDH).

**Methods:**

We retrospectively reviewed 9 consecutive AEDH patients evaluated for MMA embolization at our institution. Eight underwent embolization, and one was converted to immediate craniotomy after pre-procedural cranial CT showed rapid hematoma expansion. In the embolization subgroup, low-viscosity agents included 1:1 diluted Onyx with dimethyl sulfoxide (DMSO) (*n* = 6) and 1:9 diluted Glubran 2/ethiodized oil (*n* = 2). Primary observations included technical success, radiographic evolution, clinical outcomes, and periprocedural complications.

**Results:**

Technical success was achieved in all embolized cases (8/8). Across the eight embolized cases, low-viscosity agents reached distal dural branches without relevant proximal reflux, and no catheter adherence or catheter entrapment was observed. No procedure-related complications occurred. Follow-up imaging showed stable or resolving hematomas in all embolized patients, and none required rescue surgery for post-embolization expansion. Median discharge GCS was 15, and no new neurologic deficits were observed. In the non-embolized conversion case, immediate pre-procedural CT showed interval progression, and prompt craniotomy was performed.

**Conclusion:**

In selected AEDH patients, adjunctive MMA embolization with low-viscosity liquid embolic agents (1:1 diluted Onyx/DMSO and 1:9 diluted Glubran 2/ethiodized oil) was technically feasible with a favorable short-term safety profile and may help control bleeding and reduce the risk of hematoma expansion while balancing distal penetration and catheter control. This strategy may provide a minimally invasive treatment option for high-risk AEDH, but multicenter studies with larger retrospective case series are needed for confirmation.

## 1 Introduction

Acute epidural hematoma (AEDH) is a common neurosurgical emergency, typically resulting from traumatic injury to the middle meningeal artery (MMA) or its branches (1, 2). Emergent surgical evacuation remains standard for large hematomas with substantial mass effect. However, optimal management of small-volume or minimally symptomatic AEDH remains debated. Close observation is frequently used, but delayed expansion requiring surgery still occurs in approximately 15–23% of patients in prior series (1, 3–5). Endovascular MMA embolization has been increasingly used in selected AEDH cases to control arterial bleeding and limit further expansion. Suzuki et al. (6) first described this strategy in 2004. Subsequent reports used coils, polyvinyl alcohol (PVA) particles, and liquid embolic agents including NBCA and Onyx (7, 8). Zhang et al. reported favorable outcomes with coil embolization plus minimally invasive drainage (9), and Wang et al. (10) reported favorable outcomes using standard Onyx-18 in small AEDH. Nonetheless, standard-concentration Onyx may promote proximal plugging in some anatomies, while adhesive glue agents such as Glubran 2 carry a risk of catheter adherence or entrapment. Whether low-viscosity workflows can improve distal penetration without compromising safety remains unresolved.

Onyx (ethylene vinyl alcohol copolymer) provides deep vascular penetration and arterial-bed casting, but standard Onyx formulations (Onyx-18 or Onyx-34) have relatively high viscosity, potentially limiting access to distal dural arterioles and capillary beds where bleeding may originate. Prolonged injection also carries a recognized risk of microcatheter entrapment (11, 12). Glubran 2 is an adhesive NBCA-based glue typically mixed with ethiodized oil to adjust polymerization and radiopacity; prior neurointerventional literature indicates that NBCA/ethiodized oil concentration should be tailored to flow dynamics and catheter position (13, 14). Higher glue fractions polymerize faster and may increase proximal occlusion and catheter-adherence risk.

Based on these considerations, we adopted a low-viscosity strategy. Most embolized cases received 1:1 diluted Onyx/DMSO, and two received 1:9 diluted Glubran 2/ethiodized oil to preserve radiopacity while improving distal spread and catheter retrievability. Hung et al. (15) reported that diluted Onyx may shorten radiographic resolution time in chronic subdural hematoma, but data in AEDH remain limited. We therefore report technical details and early outcomes from a consecutive AEDH retrospective case series.

## 2 Methods

### 2.1 Patient selection

This retrospective study included 9 consecutive patients with AEDH who were initially evaluated for MMA embolization at Taicang TCM Hospital Affiliated to Nanjing University of Chinese Medicine from June 2025 to June 2026. Eight patients underwent MMA embolization, while one was converted to immediate craniotomy after pre-procedural cranial CT showed rapid hematoma expansion before transfer to the angiography suite. The study protocol was approved by the Institutional Review Board (IRB) of Taicang TCM Hospital Affiliated to Nanjing University of Chinese Medicine (Approval No. 2026015). All study procedures were conducted in accordance with the Declaration of Helsinki and relevant national ethical regulations. Because of the retrospective design, written informed consent for study participation was waived; all patients or their legally authorized representatives had provided general procedural consent.

**Figure 1:**
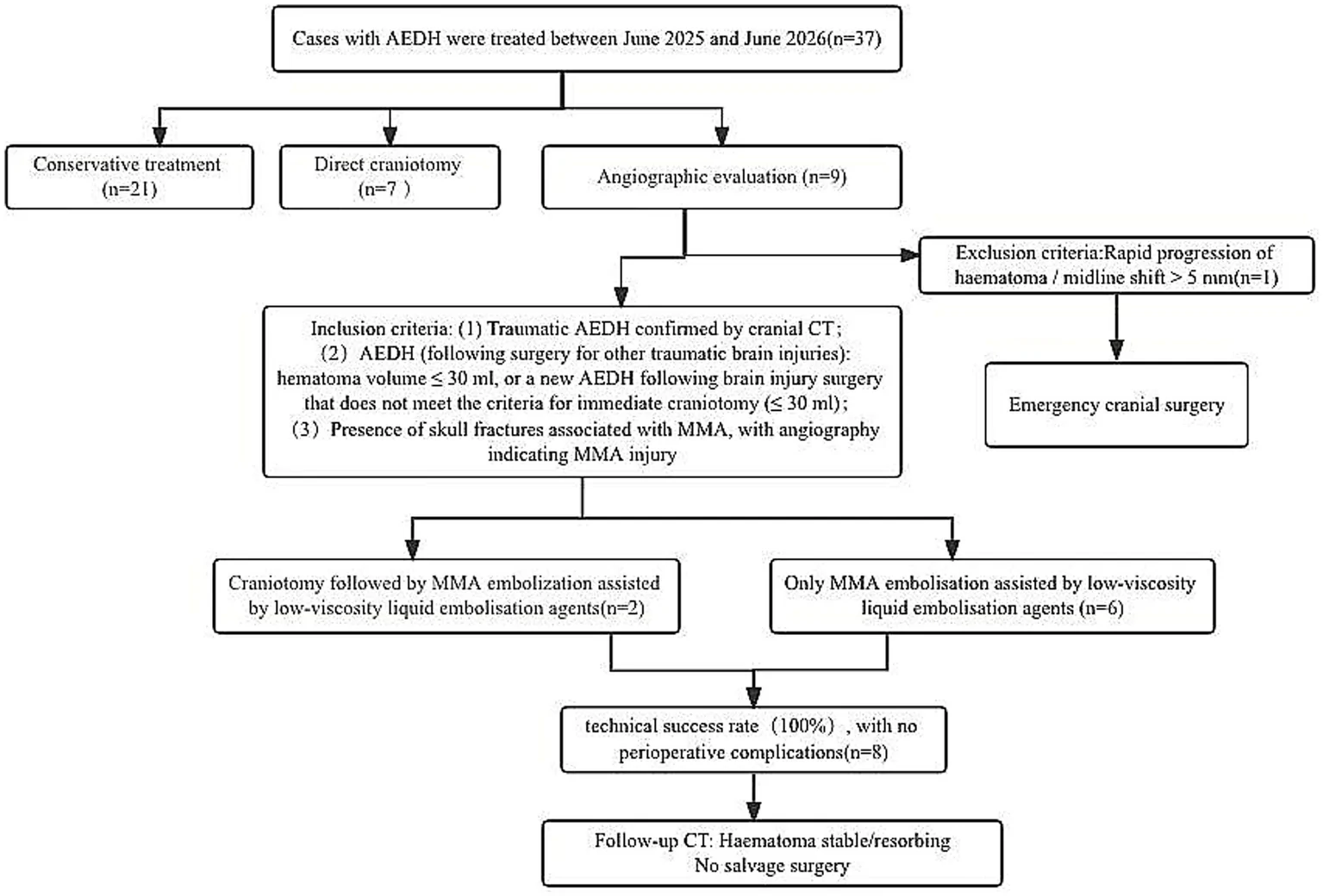
Flowchart of inclusion and exclusion criteria for this study.

Inclusion criteria: (1) traumatic AEDH confirmed by cranial CT; (2)AEDH (following surgery for other traumatic brain injuries): hematoma volume ≤ 30 mL, or a new AEDH following brain injury surgery that does not meet the criteria for immediate craniotomy (≤ 30 mL); (3)presence of skull fractures intersecting the course of the MMA with angiographic evidence of MMA injury (e.g., active contrast extravasation, pseudoaneurysm, or arteriovenous fistula) indicating a high risk of delayed expansion. Exclusion criteria:rapidly progressive neurological deterioration caused by the AEDH itself (typically a GCS score < 8) or a large hematoma (estimated at > 30 mL using the method described above); Such patients are directly referred for craniotomy. Patients with low GCS primarily caused by concomitant traumatic lesions already treated surgically could still be considered for embolization when a newly developed or persistent contralateral AEDH met the above radiographic criteria and did not require immediate second craniotomy. The patients selection flowchart is shown in Figure 1.

### 2.2 Embolic agent preparation

In 6 cases, standard Onyx (Medtronic, Irvine, CA, United States) was diluted with sterile DMSO at a 1:1 volume ratio (1 mL Onyx + 1 mL DMSO) to obtain a lower-viscosity mixture. In 2 cases, Glubran 2 (GEM, Viareggio, Italy), a modified N-butyl-2-cyanoacrylate (NBCA), was used. Glubran 2 was diluted with Ethiodized Poppyseed Oil Injection (Hengrui Medicine, China; hereafter, ethiodized oil) at 1:9 (0.1 mL Glubran 2 + 0.9 mL ethiodized oil) to optimize viscosity and radiopacity. Embolic-agent selection was not based on a rigid pre-specified algorithm; rather, it was individualized according to the procedural difficulty of distal superselection, the caliber of the MMA branch, and the need to shorten procedure time in an urgent hemorrhagic setting. When the MMA branch was relatively fine and distal microcatheterization was considered difficult, diluted Glubran 2 was generally favored because its lower-viscosity mixture was expected to disperse more readily into the distal target bed despite a less distal catheter position. When distal catheterization was more achievable and a more controlled injection was preferred, diluted Onyx was generally selected. Both mixtures were prepared immediately before embolization.

### 2.3 Endovascular procedure

All procedures in this retrospective case series were performed under general anesthesia. Because of the acute hemorrhagic setting, no systemic intravenous heparinization was administered. Instead, heparinized saline (2,000 U heparin in 500 mL normal saline) was used as a continuous flush during the procedure to maintain catheter patency while attempting to minimize the risk of hematoma enlargement. Femoral arterial access was used in all cases. A 6-French guiding catheter was positioned in the proximal external carotid artery (ECA) for diagnostic angiography. A DMSO-compatible microcatheter (e.g., Echelon-10, Medtronic) was then advanced over a microwire into the MMA trunk under roadmap guidance.

MMA angiography was then performed to confirm the exact site of MMA laceration, contrast extravasation, or pseudoaneurysm. Diagnostic ECA and superselective MMA angiography were also used to assess for dangerous anastomotic pathways (including recurrent meningeal artery–ophthalmic artery collaterals and MMA branches supplying cranial nerve microvasculature) before liquid embolization. If a dangerous anastomosis was identified angiographically, the embolization procedure would be aborted. The microcatheter dead space was then filled with DMSO (for Onyx) or D5W (for Glubran 2), and low-viscosity liquid embolization was performed under continuous fluoroscopic visualization. During injection, embolic dispersion was monitored continuously with intermittent fluoroscopic pauses; if there was any concerning tendency toward reflux or toward unintended migration into ophthalmic or intracranial arterial territories, the injection would be stopped immediately. For 1:1 diluted Onyx, a continuous slow injection was used to reach distal branches supplying the target lesion. For 1:9 diluted Glubran 2, a sandwich-style sequential technique (flush-inject-flush) was used in staged aliquots to promote distal dispersion and reduce catheter-sticking risk. After embolization, the microcatheter was aspirated and quickly withdrawn immediately upon completion of the injection to prevent catheter entrapment, and a final control angiogram via the guiding catheter confirmed occlusion of the culprit vascular lesion. Uniform procedural metric data available for all embolized patients included total diluted embolic volume (1.0 mL per case, as summarized in Table 1). However, consistent fluoroscopy duration, cumulative radiation dose, and dose-area product metrics were not uniformly archived in our retrospective electronic medical and angiography records across all 8 cases, so formal quantitative statistical analysis of radiation exposure was not feasible in the present series. All retrievable case-level procedural parameters are summarized in Supplementary Tables S1–S3.

**Table 1:** Baseline and peri-procedural characteristics of the Embolization subgroup.

Variable	Embolization subgroup (*n* = 8)
Age, years	52.8 ± 19.1
Male sex, *n* (%)	6 (75.0)
Mechanism of injury (MVA/Fall), *n*	7/1
Hematoma location (Temporal/Frontal/Occipital), *n*	5/1/2
Initial hematoma volume, mL	8.34 ± 8.03
Admission GCS, median (range)	12 (6–15)
Baseline coagulation profile	PT 10.2–15.9 s; INR 0.88–1.39; aPTT 24.3–63.9 s; platelet count 53–335 × 10^9/L
Angiographic lesion pattern	Predominantly active MMA extravasation; one illustrative case also had traumatic dAVF
Embolic agent (Onyx/Glubran 2), *n*	6/2
Dilution ratio (Onyx/Glubran 2)	1:1/1:9
Agent-selection rationale	Predominantly active MMA extravasation; one illustrative case also had traumatic dAVF
Diluted embolic volume per case, mL	1.0
Access/anesthesia	Transfemoral access under general anesthesia in all cases
Microcatheter tip position	Reconstructable in representative cases only; not systematically recorded case-by-case
Procedure time, min	89.4 ± 13.5
Technical success, *n* (%)	8 (100)
Descriptive angiographic distal embolic spread	Observed in both diluted Onyx and diluted Glubran 2 cases; not formally graded
Catheter sticking, *n* (%)	0 (0)
Procedure-related complications, *n* (%)	0 (0)
Discharge GCS, median (range)	15 (9–15)
Routine imaging time points	Immediate post-embolization CT, 24-h CT, discharge CT, and later CT when available; overall final follow-up could be CT-based or telephone-based
Overall follow-up duration, d, median (range)	42.5 (19–214)
Last documented follow-up modality (CT/telephone), *n*	6/2
Quantitative imaging outcome	No enlargement on immediate or 24-h CT in 8/8; discharge CT volume recorded as 0 mL in 8/8; among 6 patients with CT-based later follow-up, last available CT volume was 0 mL

dAVF, dural arteriovenous fistula; GCS, Glasgow Coma Scale; MMA, middle meningeal artery; MVA, motor vehicle accident. Data are presented as mean ± SD, median (range), text summary, or *n* (%), as appropriate. Table 1 includes embolized patients only (*n* = 8); one conversion-to-craniotomy case is reported separately in the manuscript text. Distal embolic spread was recorded as an intra-procedural angiographic description rather than a formal graded endpoint. Fluoroscopy time, radiation dose metrics, exact microcatheter model by case, and fully standardized device logs were not uniformly retrievable for all cases. Total diluted embolic agent volume was consistently recorded as 1.0 mL per patient across all embolization procedures. “Last available CT” refers only to the latest documented CT examination, whereas “last documented follow-up” includes both CT-based and telephone-based follow-up. Case-level volume, coagulation, and follow-up data are detailed in Supplementary Table S1.

### 2.4 Outcome measures

We assessed: (1) Technical Success: defined as complete angiographic occlusion of the target MMA branches and cessation of contrast extravasation. (2) Clinical Outcome: prevention of hematoma expansion requiring surgery, changes in GCS score from admission to discharge. (3) Radiographic Outcome: hematoma stability or regression on follow-up CT scans (performed 1–3 days post-procedure or as clinically indicated). (4) Complications: catheter entrapment, non-target embolization, stroke, access site complications, headache, or seizure.

### 2.5 Statistical analysis

Descriptive statistics were used because of the small sample size. Continuous variables are reported as mean ± SD or median (range), as appropriate, and categorical variables are presented as counts and percentages. Sex distribution was summarized as male/female counts and percentages. No formal hypothesis testing was performed.

## 3 Results

### 3.1 Patient characteristics

Among the 37 cases with AEDH treated during the study period, 21 cases were managed conservatively, 7 cases underwent craniotomy alone, and 9 cases underwent angiographic evaluation; among these, 1 case was converted to immediate craniotomy after rapid progression on pre-procedural cranial CT, 2 cases underwent craniotomy followed by adjunctive MMA embolization, and 6 cases underwent MMA embolization alone. In the embolization subgroup, 6 cases were male and 2 cases were female, with a mean age of 52.8 ± 19.1 years (range, 20–72). Mechanism of injury was motor vehicle accident in 7 cases and fall in 1 case. Hematoma location was temporal in 5 cases, frontal in 1 case, and occipital in 2 cases; all had skull fractures corresponding to the lesion site. Mean initial hematoma volume was 8.34 ± 8.03 mL (range, 2.5–27.0), and median admission Glasgow Coma Scale (GCS) score was 12 (range, 6–15). Baseline coagulation values were heterogeneous, with PT ranging from 10.2 to 15.9 s, INR from 0.88 to 1.39, aPTT from 24.3 to 63.9 s, and platelet counts from 53 to 335 × 10^9/L. Diagnostic angiography confirmed MMA-related vascular lesions (active contrast extravasation or abnormal dilation) in all 8 embolized patients. Baseline and periprocedural details are summarized in Table 1 and Supplementary Table S1 (embolized patients only).

### 3.2 Procedural outcomes

Among embolized patients, technical success was achieved in 100% (8/8), with final angiography confirming cessation of extravasation from injured MMA branches. Both 1:1 diluted Onyx and 1:9 diluted Glubran 2 were handled smoothly. In each case, a fixed total of 1 mL diluted embolic agent was sufficient to achieve complete target vessel occlusion. *Distal penetration*: In all cases, the embolic agent reached distal arteriolar and capillary MMA branches, with angiographic occlusion of the suspected bleeding territory. *Catheter removal*: No resistance was encountered during microcatheter withdrawal. There were no instances of catheter entrapment or difficult retrieval (0%). *Procedure time*: The mean procedure time was 89.4 ± 13.5 min (range: 70–110 min). Fluoroscopy duration, radiation dose metrics, exact microcatheter model by case, and fully standardized device logs were not uniformly retrievable from the current retrospective records.

### 3.3 Clinical and radiographic follow-up

Immediate post-embolization CT and the routine 24-h follow-up CT showed no hematoma enlargement in any embolized patient (0/8); hematoma volumes remained unchanged from baseline at both early time points in the tabulated case-level data. No embolized patient required rescue craniotomy. By discharge, the recorded hematoma volume was 0 mL in all 8 embolized patients. Among the 6 patients with CT-based later follow-up, the last available CT was also documented as 0 mL. The remaining 2 patients had telephone-based final follow-up after interval imaging review and therefore did not contribute a later CT time point beyond their available interval imaging. At discharge, median GCS was 15 (range, 9–15); 5 of 8 embolized patients reached GCS 15, and no new neurologic deficits were observed. The median interval from embolization to last documented follow-up, regardless of modality, was 42.5 days (range, 19–214). No dangerous-anastomosis-related or other non-target embolization complications were observed in this series. The screening-failure case underwent emergent craniotomy after rapid pre-procedural expansion to approximately 40 mL and was not included in embolization efficacy analyses.

### 3.4 Illustrative cases

**Figure 2:**
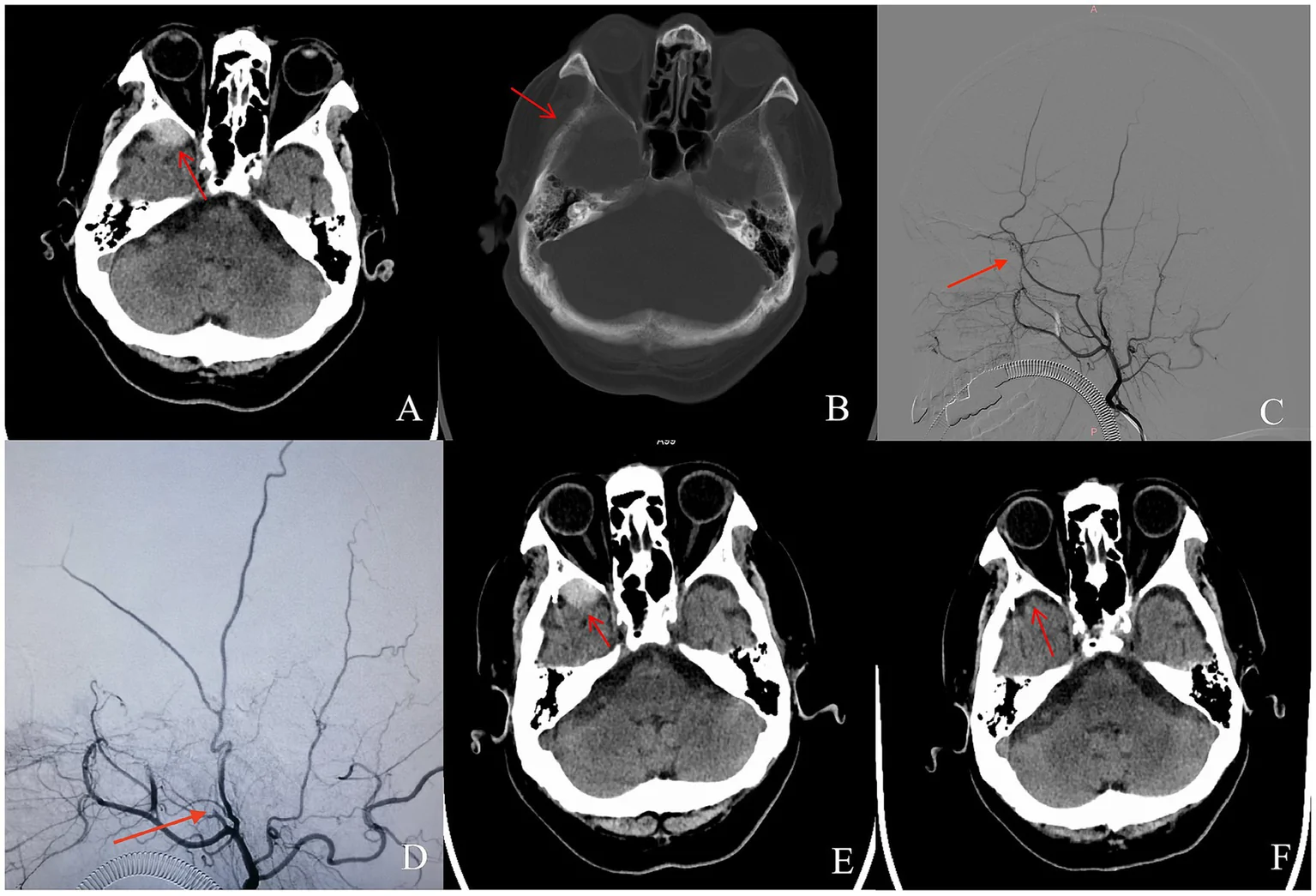
Illustrative case 1. **(A)** Axial non-contrast CT brain showing right temporal acute epidural hematoma (red arrow). **(B)** CT bone window demonstrating right temporal skull fracture (red arrow). **(C)** External carotid angiogram showing active contrast extravasation from the injured middle meningeal artery (red arrow). **(D)** Post-embolization angiogram confirming complete occlusion of the bleeding vessel (red arrow). **(E)** Immediate post-procedure CT showing resolved contrast leakage. **(F)** One-month follow-up CT with complete resolution of epidural hematoma.

*Case 1*: A 72-year-old man was admitted after a motor vehicle accident. Initial cranial CT showed a small right AEDH (approximately 3.3 mL; Figure 2A) and a right temporal bone fracture (Figure 2B). To assess the risk of further expansion, emergency cerebral angiography was performed and showed active contrast extravasation at the right MMA fracture site (Figure 2C). Immediate embolization of the MMA was then performed using diluted Onyx-18. Post-embolization angiography showed no opacification of the MMA with a visible residual stump (Figure 2D), and CT confirmed satisfactory embolization of the MMA (Figure 2E). A 1-month follow-up cranial CT showed complete resolution of the right AEDH (Figure 2F).

**Figure 3:**
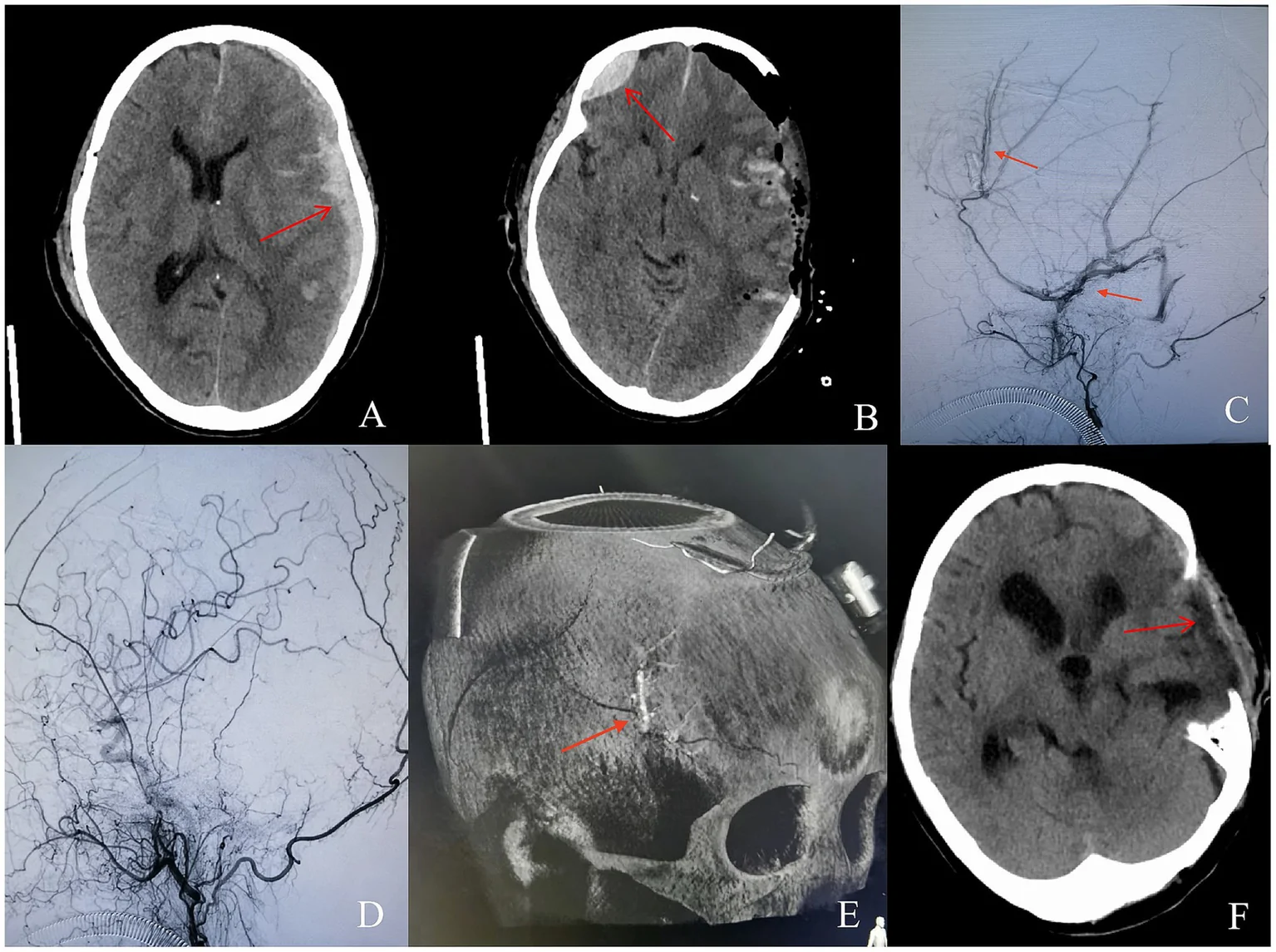
Illustrative case 2. **(A)** Admission CT showing left frontotemporal contusion and contralateral right epidural hematoma with midline shift (red arrow). **(B)** Post-craniotomy CT showing new right frontal acute epidural hematoma at fracture site (red arrow). **(C)** Angiography displaying two bleeding sites on middle meningeal artery branches (red arrows). **(D)** Post-embolization angiogram with disappearance of extravasation and traumatic dAVF. **(E)** 3D skull reconstruction showing embolic cast at fracture site (red arrow). **(F)** One-month follow-up CT with full hematoma resolution (red arrow).

*Case 2*: A 71-year-old woman was admitted after a motor vehicle accident. Initial cranial CT showed a left acute subdural hematoma with left frontotemporal contusion and a marked rightward midline shift of approximately 10 mm (Figure 3A). She underwent emergent evacuation of the left intracerebral hematoma with decompressive craniectomy under general anesthesia. Immediate postoperative CT showed a new contralateral AEDH at the right frontal skull fracture site (approximately 8.6 mL; Figure 3B). Given her advanced age, substantial prior surgical burden, and risk of further right-sided AEDH expansion, emergency cerebral angiography was performed to avoid a second craniotomy. Angiography confirmed injury of the frontal branch of the right MMA with contrast extravasation and also showed a concomitant traumatic dural arteriovenous fistula (dAVF) arising from the occipital branch;pre-embolization full external carotid angiography confirmed no patent unprotected anastomosis between MMA branches and ophthalmic artery circulation to eliminate the risk of cross-compartment embolic migration (Figure 3C). Immediate MMA embolization with Glubran 2 was performed to occlude both the bleeding point and traumatic dAVF. Post-embolization angiography showed no MMA opacification, with disappearance of both contrast extravasation and dAVF (Figure 3D), and 3D reconstruction showed a liquid embolic cast at the skull fracture site (Figure 3E). The procedure was uneventful, and 1-month follow-up cranial CT showed complete resolution of the right AEDH (Figure 3F).

**Figure 4:**
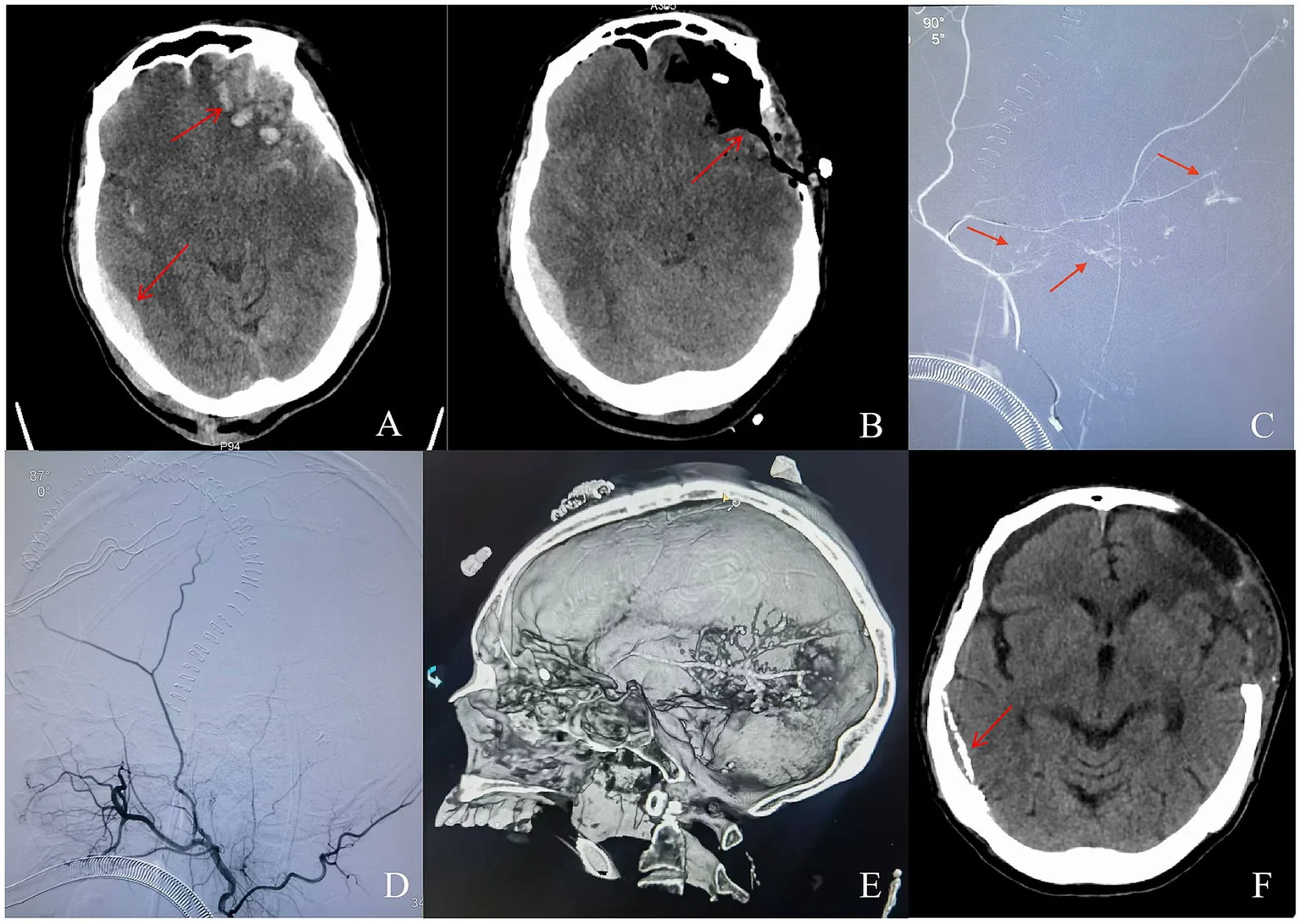
Illustrative case 3. **(A)** Initial cranial CT showed a left acute subdural hematoma with left frontotemporal contusion and a marked rightward midline shift (red arrows). **(B)** Post-decompressive craniectomy CT with residual right epidural hematoma (red arrow). **(C)** Angiography identifying MMA injury and contrast extravasation (red arrows). **(D)** Final angiogram confirming target vessel occlusion after embolization. **(E)** 3D reconstruction demonstrating distal embolic distribution along dural vasculature. **(F)** One-month follow-up CT showing complete hematoma absorption (red arrow).

*Case 3*: A 62-year-old man was admitted after a motor vehicle accident. Initial cranial CT showed a left frontotemporal-basal cerebral contusion and a right temporo-occipital AEDH with marked cisternal compression (Figure 4A). He underwent emergent left cerebral contusion evacuation and decompressive craniectomy under general anesthesia. Immediate postoperative CT showed that the left cerebral contusion had been largely cleared (Figure 4B). Given the substantial surgical burden and potential risk of further right-sided AEDH expansion, emergency cerebral angiography was performed. Angiography confirmed MMA injury at the right temporo-occipital fracture site with contrast extravasation (Figure 4C), and immediate MMA embolization with Glubran 2 was performed. The procedure was uneventful, and the diluted liquid embolic agent completely occluded the culprit branch without complications (Figure 4D). 3D reconstruction showed adequate distal dispersion of glue along the dural vascular distribution (Figure 4E). A 1-month follow-up cranial CT showed complete resolution of the right AEDH (Figure 4F).

### 3.5 Pre-procedural triage event (screening failure)

During the study period, one patient within the craniotomy-alone subgroup had initially been considered for embolization because of a small AEDH. However, repeat CT performed before transfer to the angiography suite demonstrated rapid hematoma progression to approximately 40 mL with increased mass effect, prompting immediate conversion to open craniotomy for hematoma evacuation. Because no embolization was performed, this case was treated as a pre-procedural triage event (screening failure) rather than a technical failure, and it was not included in Table 1 and Supplementary Tables S1–S3, or the embolization outcome analyses.

## 4 Discussion

In this retrospective series of selected AEDH patients, a low-viscosity liquid embolization strategy (1:1 diluted Onyx/DMSO and 1:9 diluted Glubran 2/ethiodized oil) showed good technical feasibility. In the full 9 cases retrospective case series, 8 cases underwent embolization with 100% technical success and no catheter entrapment or non-target embolization, while 1 case progressed before intervention and required immediate craniotomy. These findings reinforce that patient selection and dynamic imaging remain central to triage.

Our clinical objective is closely aligned with the single-center AEDH retrospective case series by Wang et al. (10): early endovascular hemostasis for small, fracture-associated lesions based on angiographic evidence of MMA injury. Earlier studies by Suzuki et al. (6) and Peres et al. (16) established the procedural background for embolization in carefully selected AEDH, largely using more conventional embolic materials such as NBCA, PVA particles, and gelatin sponge. Wang et al. (10), by contrast, described a more contemporary single-center experience using standard-concentration Onyx-18 and therefore represents a more relevant comparator for conventional liquid embolic therapy. Within this literature context, the present study is best interpreted as a technical case series exploring a low-viscosity liquid embolic workflow rather than as comparative evidence that this strategy is superior to standard Onyx-18, conventional NBCA mixtures, or coil embolization. Additional case-level support under strict neurologic and imaging surveillance has also been reported by Oláh et al. (17). Preoperative CTA may help identify contrast leakage, pseudoaneurysm, or other imaging features suggestive of ongoing MMA-related bleeding before catheter angiography, although this workflow was not used systematically in our retrospective case series.

Mechanistically, lower viscosity may improve distal dispersion and reduce injection resistance. In our practice, this consideration was most relevant when the MMA branch was fine, distal superselection was difficult, and the suspected bleeding territory extended beyond a single proximal injury point. However, this remains a procedural rationale rather than a demonstrated mechanistic advantage. Onyx offers nonadhesive, controllable injection, whereas Glubran 2 requires strict anti-reflux discipline because of adhesive behavior; with low-concentration mixtures, 5% glucose flushing, and sequential flush-inject-flush technique, safe delivery remained achievable in our retrospective case series (18–21). Regarding the radiopacity of the embolic mixture, while a 1:1 dilution of Onyx with DMSO theoretically halves the concentration of suspended micronized tantalum, we found that the mixture remained clearly visible under standard high-quality fluoroscopy in our clinical practice. This sufficient radiopacity allowed for adequate monitoring of the forward progression of the embolic agent. Nevertheless, to proactively prevent any unrecognized catheter adherence or entrapment—a valid theoretical concern when altering embolic viscosity—high-quality digital subtraction roadmap guidance was strictly utilized during the injection phase. The injection was performed slowly with frequent brief pauses to verify the absence of retrograde reflux along the catheter shaft. Furthermore, microcatheter removal was performed promptly upon completion of the intended delivery before significant proximal polymerization could occur. With these standardized precautions, no catheter sticking was observed in this series. Notably, two patients with high-risk contralateral AEDH after decompressive craniectomy (including one with traumatic dAVF) were controlled in a single endovascular session, avoiding repeat craniotomy.

While low-viscosity embolic mixtures enable deeper distal penetration to seal remote bleeding arteriolar lesions, their enhanced flowability theoretically increases the risk of uncontrolled distal embolic migration across tiny meningeal-intracranial anastomoses, most notably the recurrent meningeal artery–ophthalmic artery collateral pathway demonstrated in case 1 (Figure 2). Two severe procedure-related complications merit careful consideration: non-target embolization of the ophthalmic artery, which can lead to retinal ischemia, permanent vision loss, or ophthalmoplegia; and occlusion of microvessels feeding cranial nerves V and VII, potentially triggering trigeminal neuropathy or facial weakness. To mitigate these risks, we implemented three tiered safety measures: first, complete pre-intervention external carotid and superselective MMA DSA screening to rule out patent dangerous anastomoses; second, slow, intermittent embolic injection with frequent fluoroscopic pauses to track forward embolic spread; third, immediate microcatheter withdrawal upon target vascular occlusion to avoid delayed retrograde reflux. No ophthalmic or cranial nerve ischemic complications were observed in our 8 embolized patients during short-term follow-up. Nevertheless, our small sample size precludes definitive risk quantification, and larger cohorts are needed to stratify anastomosis-related adverse event rates for diluted low-viscosity embolic agents.

Several limitations should be noted. First, this was a small, single-center, retrospective case series with only 8 of 37 AEDH patients undergoing embolization. No formal comparison across treatment pathways was performed, limiting causal inference. Embolic-agent selection relied on intraprocedural judgment rather than a standardized protocol. Second, the study did not directly compare the low-viscosity workflow with conventional liquid embolic agents (e.g., standard-concentration Onyx-18). Thus, any potential benefits in distal distribution, hematoma control, or clinical outcomes remain inferential. Baseline coagulation profiles were highly heterogeneous, and hematoma volume was estimated by the ABC/2 method (less accurate than software-assisted volumetry) (22, 23), both of which may confound results. Additionally, key procedural data (lesion subtype, microcatheter tip position, fluoroscopy time, radiation dose) were incomplete due to the retrospective design. Follow-up was short (median 42.5 days) and methodologically inconsistent, precluding assessment of delayed complications. Technical risks (non-target embolization, dangerous anastomosis migration) persist, and one patient deteriorated before embolization requiring urgent craniotomy. Fourth, the 1:1 Onyx/DMSO dilution protocol used in six patients is an off-label modification without established neurointerventional consensus, and the long-term vascular toxicity associated with increased DMSO exposure remains incompletely characterized; our small sample cannot exclude rare DMSO-mediated adverse vascular events. This technique should be considered a selective adjunct, not a replacement for timely decompressive surgery.

## 5 Conclusion

In this single-center case series of carefully selected small-to-moderate, high-risk AEDH patients, adjunctive MMA embolization with low-viscosity liquid embolic agents was technically feasible in the emboli zed subgroup, short-term observation showed no procedure-related complications and no rescue craniotomy for delayed expansion. These preliminary findings support further study of this minimally invasive strategy in selected patients, but they do not establish efficacy in controlling bleeding or preventing hematoma progression. Larger multicenter prospective studies are needed to validate safety, clarify clinical utility, and refine patient-selection criteria and procedural workflows.

## Data Availability

The datasets presented in this article are not readily available because of ethical and privacy restrictions. Requests to access the datasets should be directed to the corresponding authors.
