# Supplementary material for: Adjunctive middle meningeal artery embolization with low-viscosity liquid embolic agents for acute epidural hematoma: technical considerations and a case series
Source: Front Neurol. 2026 Aug 12;17:1896356. doi: 10.3389/fneur.2026.1896356 (PMC13506484; doi:10.3389/fneur.2026.1896356)
Supplement: Supplementary file 1 [file Table_1.DOCX]

# Supplementary Material

**Supplementary Table S1-3. Case-Level Baseline, Coagulation, Volume Dynamics, and Follow-Up Data of the Embolization Subgroup**

### **Table S1**: Baseline Demographics and Clinical Characteristics

| Case No. | Age (yr) / Sex | Mechanism | Location | Adm. GCS (E-V-M) | PT (s) | INR | aPTT (s) | PLT (×10^9/L) |
| --- | --- | --- | --- | --- | --- | --- | --- | --- |
| 1 | 72 / M | MVA | Temporal | 15 (4-5-6) | 12.9 | 1.12 | 25.2 | 53 |
| 2 | 71 / F | MVA | Frontal | 6 (1-1-4) | 15.9 | 1.39 | 63.9 | 125 |
| 3 | 62 / M | MVA | Occipital | 7 (1-2-4) | 10.2 | 0.88 | 25.0 | 165 |
| 4 | 71 / M | MVA | Temporal | 12 (3-4-5) | 11.2 | 0.97 | 25.6 | 117 |
| 5 | 20 / M | MVA | Occipital | 15 (4-5-6) | 12.5 | 1.09 | 27.8 | 165 |
| 6 | 42 / F | MVA | Temporal | 12 (3-4-5) | 11.7 | 1.02 | 24.5 | 271 |
| 7 | 41 / M | MVA | Temporal | 11 (3-3-5) | 11.6 | 1.01 | 29.9 | 284 |
| 8 | 43 / M | Fall | Temporal | 15 (4-5-6) | 10.8 | 0.94 | 24.3 | 335 |

### **Table S2**: Procedural Details and Hematoma Volume Dynamics

| Case No. | Embolic Agent (Ratio) | Proc. Time (min) | Distal Spread | Cath. Sticking | Complications | Base. Vol. (mL) | Imm. Vol. (mL) | 24h Vol. (mL) |
| --- | --- | --- | --- | --- | --- | --- | --- | --- |
| 1 | Onyx (1:1) | 70 | Branch dist. | No | None | 3.3 | 3.3 | 3.3 |
| 2 | Glubran 2 (1:9) | 90 | Meningeal accum. | No | None | 8.6 | 8.6 | 8.6 |
| 3 | Glubran 2 (1:9) | 95 | Meningeal accum. | No | None | 10.0 | 10.0 | 10.0 |
| 4 | Onyx (1:1) | 110 | Branch dist. | No | None | 27.0 | 27.0 | 27.0 |
| 5 | Onyx (1:1) | 100 | Branch dist. | No | None | 5.0 | 5.0 | 5.0 |
| 6 | Onyx (1:1) | 80 | Branch dist. | No | None | 7.3 | 7.3 | 7.3 |
| 7 | Onyx (1:1) | 75 | Branch dist. | No | None | 2.5 | 2.5 | 2.5 |
| 8 | Onyx (1:1) | 95 | Branch dist. | No | None | 3.0 | 3.0 | 3.0 |

### **Table S3**: Discharge and Follow-up Outcomes

| Case No. | Disch. Vol. (mL) | Last F/U Vol. (mL) | Disch. GCS (E-V-M) | F/U Modality | F/U Duration (days) |
| --- | --- | --- | --- | --- | --- |
| 1 | 0 | 0 | 15 (4-5-6) | CT | 30 |
| 2 | 0 | 0 | 9 (3-2-4) | CT | 31 |
| 3 | 0 | 0 | 14 (4-4-6) | CT | 46 |
| 4 | 0 | 0 | 11 (3-3-5) | CT | 118 |
| 5 | 0 | N/A* | 15 (4-5-6) | Telephone | 39 |
| 6 | 0 | 0 | 15 (4-5-6) | CT | 214 |
| 7 | 0 | N/A* | 15 (4-5-6) | Telephone | 19 |
| 8 | 0 | 0 | 15 (4-5-6) | CT | 56 |

Abbreviations: Adm., admission; aPTT, activated partial thromboplastin time; Base., baseline; Cath., catheter; CT, computed tomography; Disch., discharge; F/U, follow-up; GCS, Glasgow Coma Scale; Imm., immediate; INR, international normalized ratio; MVA, motor vehicle accident; PLT, platelet count; Proc., procedure; PT, prothrombin time; Vol., volume.

- For patients whose final follow-up was conducted via telephone, the last available imaging was the discharge CT (showing 0 mL). Distal spread was recorded as an intra-procedural angiographic description rather than a formal graded endpoint.
